# Mechanistic Indicators of Childhood Asthma (MICA) Study: piloting an integrative design for evaluating environmental health

**DOI:** 10.1186/1471-2458-11-344

**Published:** 2011-05-19

**Authors:** Jane Gallagher, Edward Hudgens, Ann Williams, Jefferson Inmon, Scott Rhoney, Gina Andrews, David Reif, Brooke Heidenfelder, Lucas Neas, Ronald Williams, Markey Johnson, Haluk Özkaynak, Stephen Edwards, Elaine Cohen Hubal

**Affiliations:** 1 National Health and Environmental Effects Research Laboratory, U.S. Environmental Protection Agency, Research Triangle Park, North Carolina, 27711, USA; 2 National Center for Computational Toxicology, U.S. Environmental Protection Agency, Research Triangle Park, North Carolina, 27711, USA; 3 U.S. Environmental Protection Agency, National Exposure Research Laboratory, Research Triangle Park, North Carolina, 27711, USA

## Abstract

**Background:**

Asthma is a common complex disease responsible for considerable morbidity and mortality, particularly in urban minority populations. The Mechanistic Indicators of Childhood Asthma study was designed to pilot an integrative approach in children's health research. The study incorporates exposure metrics, internal dose measures, and clinical indicators to decipher the biological complexity inherent in diseases such as asthma and cardiovascular disease with etiology related to gene-environment interactions.

**Methods/Design:**

205 non-asthmatic and asthmatic children, (9-12 years of age) from Detroit, Michigan were recruited. The study includes environmental measures (indoor and outdoor air, vacuum dust), biomarkers of exposure (cotinine, metals, total and allergen specific Immunoglobulin E, polycyclic aromatic hydrocarbons, volatile organic carbon metabolites) and clinical indicators of health outcome (immunological, cardiovascular and respiratory). In addition, blood gene expression and candidate SNP analyses were conducted.

**Discussion:**

Based on an integrative design, the MICA study provides an opportunity to evaluate complex relationships between environmental factors, physiological biomarkers, genetic susceptibility and health outcomes.

**Project approval:**

IRB Number 05-EPA-2637: The human subjects' research protocol was reviewed by the Institutional Review Board (IRB) of the University of North Carolina; the IRB of Westat, Inc., the IRB of the Henry Ford Health System; and EPA's Human Subjects' Research Review Official.

## Background

Asthma is a chronic disease of the airways involving airway inflammation, variable obstruction, hyper responsiveness, and respiratory symptoms. Asthma is a significant problem worldwide that causes substantial social impacts and costs to public and private health systems [1]. Asthma is poorly understood in part because of the myriad of complex genetic and environmental components which influence both the development and exacerbation of asthma.

Understanding the complex relationships between environmental exposures and health outcomes such as asthma requires consideration of a wide range of factors--both extrinsic (e.g., ambient air quality) and intrinsic (e.g., genotypic) that must be integrated to assess cumulative risk and to support epidemiological studies investigating gene-environment interactions. As understanding of how to assess and mitigate health risks resulting from exposures to individual environmental pollutants improves, environmental health scientists are turning their attention toward characterizing relationships between multiple environmental factors and complex diseases. Although the association of genetic and environmental factors in the development of disease has long been recognized, promising tools for characterizing global human genetic variation have only recently been developed [2]. The study of the role of genetic variation in human response to environmental exposures, "ecogenetics" [3], focuses on identifying markers of susceptibility and characterizing the modification of risk in susceptible individuals. Recent technological advances have led to the development of toxicogenomics which examines the effects that chemicals have on living organisms and/or the environment using genomic, proteomic, and metabonomic methods [4]. These emerging tools in molecular biology provide the potential for development of cellular and molecular indicators of exposure and health status that can be used to assess the vulnerability of humans to environmental stressors.

In the Mechanistic Indicators of Childhood Asthma study (MICA), biomonitoring and other health data are used to characterize potential exposures and asthma-related health outcomes. By considering an array of metrics across the exposure-outcome continuum, this framework begins to address the multi-factorial nature of environmental disease and cumulative risk. Because it is unlikely that a single "ideal" biomarker (a single measure with all the important characteristics for relating health outcomes with a particular exposure) will be identified, an array of biomarkers is measured to evaluate the relationships.

An overarching hypothesis of MICA is that genomic data viewed together with a spectrum of exposure, effects, clinical and susceptibility markers: a) increases the sensitivity needed to define exposure-response-effects relationships, and b) provides mechanistic insight useful from a clinical and environmental health context. Specifically this rich data set will also be used to:

• Segregate asthma phenotypes and develop hypotheses related to underlying biological mechanisms through the application of multidimensional statistical and knowledge-based approaches

• Identify panels of biomarkers from the array of MICA exposure to health outcome covariates for future application in large population studies by evaluating reliability, predictive value, sensitivity, specificity, affordability, and applicability.

• Identify impact of exposures on key pathways identified from the gene expression data.

• Evaluate genetic variants previously associated with asthma outcomes in the MICA study population.

• Estimate individual and combined effects of environmental exposures and genetic susceptibility in determining the likelihood of childhood asthma related outcomes.

## Methods/Design

The study design and protocols were approved by the Institutional Review Boards at Henry Ford Health System (Detroit, MI), Westat Inc. (Rockville, MD), and the University of North Carolina at Chapel Hill (Chapel Hill, NC - US EPA's IRB of record). Written consent was obtained from guardians, and written assent was obtained from each child, with an oral review of both consent and assent prior to study enrollment. The (MICA) study was conducted by the United States Environmental Protection Agency between November 2006 and January 2007 in Detroit, Michigan. MICA is a cross-sectional study based on a stratified sample of children using two strata: children with asthma and children without asthma, selected in an approximately 1:1 ratio. A total of 205 children age 9-13 years old participated in the clinical study.

**Figure 1:**
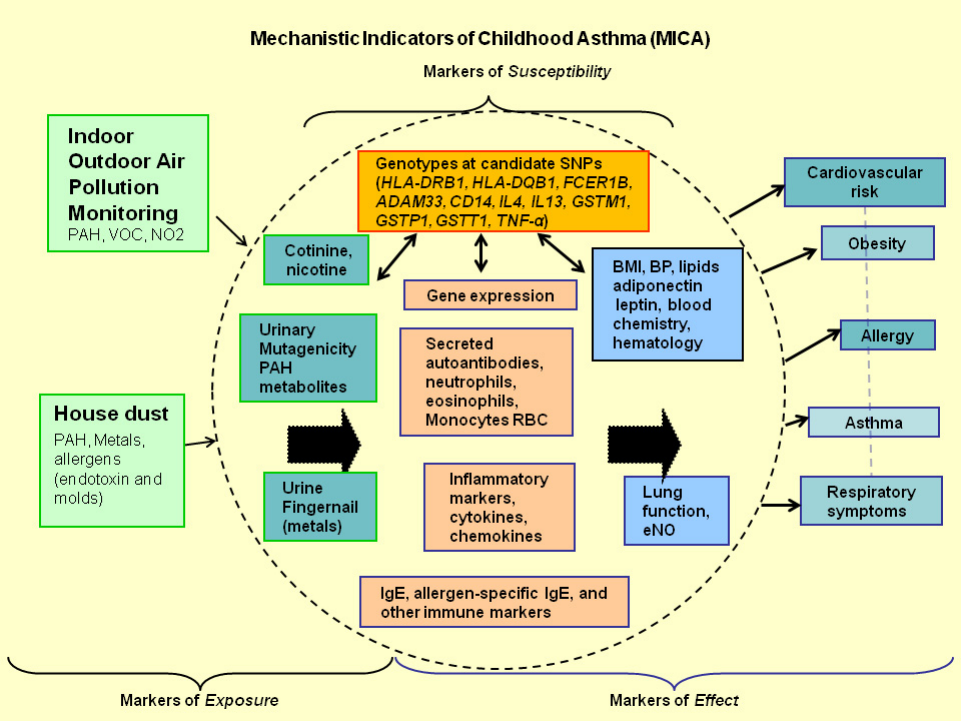
**The overall study design includes exposure, biomarkers of exposure, clinical indicators, genomic data (blood gene expression and SNP) and health status indicators**.

The integrated study design and framework for MICA is shown in Figure 1. The MICA design focuses on environmental exposures, susceptibility, asthma and other health measures, including risk factors associated with obesity and cardiovascular disease. Information on a wide range of risk factors relevant to asthma and asthma exacerbations were characterized through collection of exposure (indoor and outdoor air pollution monitors and home vacuum dust) lung function tests and biological and clinical indicators measured in blood, urine, and finger nails as shown in additional file 1, Table 1.

### Subject selection and recruitment

MICA is a nested study within the EPA's Detroit Children's Health Study (DCHS) that identified 1,157 children who completed health questionnaires as well as clinical examinations of lung function and exhaled breath [5]. From among these participants, a total of 205 non-asthmatic and asthmatic children aged 9-13 years were recruited into the MICA study at the Henry Ford Health System (HFHS) clinic in Detroit, Michigan over a 10-week time period (October 25, 2006 - January 6, 2007). Recruitment contact was made by mail and telephone by Henry Ford Health System (HFHS) and involved repeated contacts in order to schedule the clinical visit. HFHS identified asthmatic (100) and non-asthmatic (100) children.

#### Inclusion Criteria

Children aged 9 to 12 years residing in the communities of Detroit, Dearborn, Highland Park, or Hamtramck and who are served by the Henry Ford Health System were eligible for selection into the MICA study. Subjects were recruited without regard to sex or ethnicity. A child was considered asthmatic if the clinical record showed one or more asthma-related emergency department visits, two or more asthma-related outpatient visits, or two or more asthma-related medications. From the parental questionnaire, a child was considered asthmatic with a parental report of a physician's diagnosis of asthma.

#### Exclusion Criteria

Any child with a history of respiratory illness in the last two weeks, or who had ever smoked five or more cigarettes, or who had been a carrier of a communicable disease was excluded from the pulmonary function examination. No child was excluded from participating in the pulmonary examinations due to any chronic, non-infectious respiratory problems or environmental exposures. However, to control for potential confounding by rare conditions with strong pulmonary effects, the analysis of the environmental associations excluded children with a history of cystic fibrosis, chest operation, heart conditions, or who received oxygen for more than two weeks after birth or at home. Children with any of these conditions, with acute respiratory illnesses, or active smokers were excluded from the pulmonary exams.

#### Measurement Methods

##### Questionnaire

A detailed questionnaire regarding the child's medical history and family history as well as the child's home environment was obtained. In addition, a short questionnaire was obtained on the day of the clinic visit to assess the child's recent medical history including smoking history, time-activity patterns and summary of the child's daily diet.

##### Participant-Based Residential Environmental Sampling

All MICA study participants were asked to bring in a used vacuum cleaner bag to serve as a sample of dust from the home environment. In addition, the parents of a subset of asthmatic and non-asthmatic children (n = 100) also completed a participant-based collection of indoor and outdoor air samples from the children's homes that focused on passively measured nitrogen dioxide (NO_2_), polycyclic aromatic hydrocarbons (PAH) and volatile organic compounds (VOC) as shown in Figure 1 and additional file 1, Table 1.

##### Lung Function, Expired Nitric Oxide, Serum Based Allergen Sensitivity Tests

Spirometry was performed with an automated spirometer consistent with American Thoracic Standards. Lung function measurements were repeated 3-8 times and exhaled nitric oxides test were repeated 3-5 times for accuracy. Breathing tests (forced expiratory volumes and expired nitric oxide) were recorded on chart data sheets. Total IgE and allergen specific IgE levels for a multi-allergen screen were quantified for each subject's blood. The multi-allergen screen detects the presence (positive/negative) using a panel of at least 15 common aeroallergens and 5 common food allergens.

##### Height, Weight, Blood Pressure and Blood Oxygen

Height and weight measurements were obtained. A trained technician measured the child's blood pressure with oscillometric digital blood pressure monitor and repeated the measure three times. Blood oxygen was measured with a portable pulse oximeter.

##### Blood Sample Collection

Blood from non-fasting children (approximately 50 mls) was drawn into10 separate Vacutainer™ tubes in the following order: 5 mls into 2-2.5 ml/tube (PAXgene Qiagen, Valencia, CA, USA); 15 mls into 3-6 ml/tube serum separator tubes; 2 mls into 2-3 ml/tube minicollector EDTA-containing tubes; and 2, 9, and 4 mls into 3 sodium citrate tubes. HFHS conducted analyses of CBC, Pb and Hg using standard methods as mandated by CLIA (Clinical Laboratory Improvement Amendments). All other measurements in blood are listed in additional file 1, Table 1.

##### Urine and Fingernail Collection

Urine was collected from each child in a container that was fitted to the top of the toilet lid. Each child was also asked to clip his/her fingernails and/or toenails which were placed in two separate plastic Ziploc bags. Measurements in urine and nails are listed in additional file1, Table 1.

#### Molecular Markers

##### Single Nucleotide Polymorphisms (SNPs)

SNP genotypes were selected as candidate SNPs (HLA-DRB1, HLA-DQB1, FCER1B, ADAM33, CD14, IL4, IL13, GSTM1, GSTP1, GSTT1, and TNF). The rationale for selection of the 11 specific genes and relevant SNPs is based on relevant studies in which associations between asthma and specific genotypes were evaluated. The SNPs were selected by querying public databases for polymorphisms showing variation in African American (AA) samples, since that race was overrepresented. For genes with no AA variable SNPs in the databases, we selected based on studies reported in the literature. To rank the available SNPs within each gene, we used a simple "weight of evidence" summation.

##### Whole Blood Gene expression

Peripheral blood samples collected in PAXgene blood RNA tubes (Qiagen, Valencia, CA, USA), were inverted several times to mix, held at room temperature for 24 hours to lyse red blood cells, and then stored at -80°C. The PAXgene blood tubes contain reagents to stabilize RNA in blood. Frozen blood samples were transported to the US EPA on dry ice and stored at -80°C until RNA was isolated. Total RNA was isolated using DNase treatment. RNA quality was checked using an Agilent Bioanalyzer. 2.5-5.0 mg of each sample was sent to Expression Analysis (Durham, NC) for cDNA target generation and hybridization to Affymetrix Human U133 Plus 2.0 whole genome arrays.

#### Database/Data Analysis

##### Database

The MICA database is derived from the MICA data collection and samples. The database is maintained and updated in Access (Microsoft) and SAS (version 9.1, Cary, NC) databases and contains anonymized MICA Study participant data and results of sample analyses. All personal information provided by the subject to the investigators and all information that is collected about the subject by the investigators' subject identifiers have been stripped from all data records. Analytic data is identified through subject ID numbers. The Database lists the names of the MICA data tables and provides a brief description of the contents of those tables. Additional file 1, Table 1 lists the named variables for the MICA data tables.

##### Sample Size/Power calculations

MICA allows for the comparison of multiple markers of exposure, effects, and susceptibility in asthmatic and non asthmatic children. Power calculations were performed prior to data collection to estimate the sample size needed to address the major aim of the gene expression study: identification of biomarkers/factors associated with differential asthma susceptibility. The calculations were performed using the Two Sample Means option in the SAS POWER procedure. Estimates of the sample size needed to detect a 2-fold difference in mean group (i.e. asthmatics versus non-asthmatics) expression with 80% power and a stringent false-positive (alpha) rate of 1/2500 were performed. All calculations assumed the same sample proportions and measurement variance (expressed as standard deviations) in each group. The standard deviations for the log_2 _transformed measurements were varied from 0.25 to 1.0, which is in-line with observed standard deviation:mean ratios in other studies. Even with the stringent false-positive collar of 0.0004, under the most pessimistic variance scenario, the estimated sample size needed for each group was 42. The final study used a sample size of roughly twice this estimate (100 per group) in order to account for violation of the assumptions underlying our power calculations and the possibility of subgroups within the clinical asthma classes.

##### Data Analysis

Multivariate linear regression models will be used to test the association of exposures with internal measures of exposure, effect and susceptibility. Adjustments will be made for confounding and effect modifying exposures. Curves will be generated for non-asthmatics and asthmatics and tested for statistical differences between them. Separate curves will be drawn based on differences in individual polymorphisms, atopic status using individual and combined indexes for exposure and effects markers.

##### Statistical Analysis of Gene expression and SNP data

Exposure biomonitoring and clinical data from asthmatic and non-asthmatic children will be viewed in the context of markers of effect and susceptibility. The computational analysis needed to ultimately integrate the data across the sources to outcome paradigm is enormous, requiring data access and management, data mining, data interchange and data reduction. Since neither covariate data nor gene expression data alone unambiguously separates asthmatics from non-asthmatics, correlation between covariates and gene expression will be considered in order to provide context for each type of data. It is anticipated that incorporation of the covariate information will enhance the functional significance of gene clusters, and knowledge of covariate-association(s) should provide a biological context with which to interpret gene expression results.

##### Data analysis method(s) applied will include techniques from various fields

• Traditional statistics: linear regression, logistic regression, ANOVA, linear discriminant analysis.

• Machine learning: recursive partitioning trees, bootstrap aggregation (bagging) techniques, evolutionary computation-optimized classifiers, multifactor dimensionality reduction, random forests.

• Bioinformatics: protein interaction databases, knowledge (literature) mining tools, biological pathway database and inference software.

• Graphical approaches: cluster diagrams, expression "heat" maps, dendrograms, overlaid scatter plots (both exploratory and summary), distributional "violin" plots, regression plots.

## Discussion

Past approaches for designing studies and evaluating collected data have tended to focus on a limited number of environmental factors and/or measures of outcome and are rarely conducted with children. The (MICA) study design represents an integrative approach in children's health research that incorporates exposure metrics, internal dose measures, clinical indicators and blood gene expression measures to decipher the biological complexity inherent in diseases with etiology related to complex gene-environment interactions. The coordinated collection of these complimentary data provides a platform for applying systems approaches to evaluate complex relationships between environmental factors, physiological biomarkers, and health outcomes. The study can be used to inform the design of future environmental health studies and facilitate the interpretation of study results for public health decision making.

The analysis of transcriptional responses in blood coupled with biomonitoring data should provide mechanistic insight into the biological pathways that are perturbed in response to single chemicals or complex mixture exposures. Bioindicators for these biological pathways can then be incorporated into future biomonitoring studies to link the biomarkers of exposure to a relevant apical endpoint.

While MICA is an asthma study, this rich data set can be explored for relationships between early indicators of cardiovascular disease, obesity, and asthma and to demonstrate the complex nature of childhood health and environmental disease. For example, a myriad of disease risk factors/modifiers often compromise the evaluation of a single disease. The risk of developing heart disease increases to over six fold [6] with the combination of the following three risk factors: high blood pressure, high cholesterol and diabetes. Obesity is associated with numerous co-morbidities such as cardiovascular diseases, type 2 diabetes, hypertension, and certain cancers [7]. Metabolic syndrome as described by Wilson et al. [8] occurs as a constellation of obesity, hypertension, dyslipidemia, and insulin resistance leading to diabetes. MICA blood gene expression data, viewed in the context of phenotypic data relevant to asthma, cardiovascular risk, and obesity, may provide insight into exposure assessment, pathophysiology of asthma and early indications of cardiovascular disease and metabolic syndrome.

MICA provides an opportunity to pilot the application of a more holistic analytical approach to improve the interpretation of biomarker data to advance both mechanistic based toxicology and risk assessments of chemical (single, multiple, and complex mixtures) exposures. Whether and to what extent these exposures impact such a distinctive and diverse set of health outcomes such as asthma, cardiovascular disease and metabolic syndrome, are central to the MICA study objectives. Ultimately, the goal is to identify a specific and sensitive panel of molecular and traditional environmental and clinical markers from the myriad of indicators included in the MICA study design, and in doing so, gain mechanistic understanding of potential gene environment interactions that will inform design of future clinical and epidemiological studies in humans.

## Abbreviations

AA: African American; BP: Blood pressure; BMI: Body mass index; BUN: Blood urea nitrogen; CCE: Cladosporium cladosporiodes; CLIA: Clinical lab improvement methods; DCHS: Detroit children's health study; feno: forced expired nitric oxide; HDM: House dust mite extract; HFHS: Henry Ford Health System; IgE: Immunoglobulin E; IRB: Institutional Review Board; MCV: Mean cell volume; MCH: Mean cell hemoglobin; MICA: Mechanistic Indicators of Childhood Asthma Study; MACA: Metarhizium anisopliae; LDL, HDL VLDL: Low, high and very low density lipoprotein; MM1 and MM2: Mold Mix 1 and 2; NO2: Nitrogen dioxide; OHNAP1and 2-hydroxy-naphthalene; OHFLU: 2-hydroxy fluorine; OHPHE: 1-,2-&3-,4-and 9-hydroxy phenanthrene; OHPYR:1-hydroxy-pyrene; NO2: Nitrogen dioxide; PAH: Polycyclic aromatic hydrocarbons; PCE: Penicillium hhrysogenum; RDW: red blood cell distribution width; VOC: Volatile organic carbons; SCE: Stachybotrys chartarum; ALT (SGPT): Serum Glutamic pyruvic transaminase; SNPs: Single-nucleotide polymorphisms.

## Competing interests

The research described in this paper was funded wholly by the US Environmental Protection Agency and subjected to review by the National Health and Environmental Effects Research Laboratory and approved for publication. Approval does not signify that the contents necessarily reflect the views and policies of the Agency nor does mention of trade names or commercial products constitute endorsement or recommendation for use. The authors declare they have no competing or financial interests.

## Authors' contributions

JG; Principal investigator. ECH, JG; prepared draft and revisions to manuscript; developed visioning framework for assessing linkages between exposure and internal measures and health status. LN; Principal Investigator Detroit Children's Health Study. MJ, HO, RW; designed and implemented the indoor and outdoor air measurement portion of the study. AW, EH; field and study coordinators. SR, JI; prepared protocols for biological and vacuum dust sample collection and oversaw the collection, processing and shipment of samples. DR, SE, BH; collection of and study design for the blood gene expression arrays and SNP selections. GA; MICA questionnaire development and age appropriate asthma presentation explaining the study. All authors read and approved the final manuscript.

## Pre-publication history

The pre-publication history for this paper can be accessed here:

http://www.biomedcentral.com/1471-2458/11/344/prepub

## Supplementary Material

Additional file 1 **Table 1 The exposure, biomarkers, clinical indicators, and genetic variables measured in MICA**. Click here for file
