# Supplementary material for: Mechanistic Indicators of Childhood Asthma (MICA) Study: piloting an integrative design for evaluating environmental health
Source: BMC Public Health. 2011 May 19;11:344. doi: 10.1186/1471-2458-11-344 (PMC3112137; doi:10.1186/1471-2458-11-344)
Supplement: Additional file 1 — Table 1 The exposure, biomarkers, clinical indicators, and genetic variables measured in MICA. [file 1471-2458-11-344-S1.PDF]

Classification		Variable Names		
CLINICAL	**Asthma**	Asthma (PastWeek)	Dr diagnosis of asthma	Current asthma (12 months)
		Asthma by Med Use	Asthma Symptom	
	**Clinic**	Height	Mean diastolic	BMI
		Pulse	Mean systolic	O_2 Saturation
	**Lung Function**	Exp Flow	Fev1	Fev5 Fvc
		Fef25_75	Fev1/ Fvc	Fvc
		Fev5	Feno	Pef
	**Allergen Screen**	Food screen(5 food allergens)		Phadiatop(15aeoallegens)
	**Blood Chemistry**	Calcium	Creatinine	Osmolality
		Chloride	Globulin Total	C-reactive protein
		Potassium	LDH	BUN_Creatinine
		Total Protein	AST(SGOT)	Calcium
		Phospholipid C	GGT	Chloride
		Iron	A1C %	A to G Ratio
		Phosphorus	Ketones	Ketones
		Fibrinogen	Ascorbic Acid	Ferritin
		Chloride	ALT(SGPT,SGPT)	Glucose
		Alkaline Phosphatase	Bilirubin Total	Albumin
	**Inflammatory**	Nitrotyrosine	Total antioxidant status	Uric Acid
		ROS	IL4_I	UIBC
	**Lipids**	HDL	Total cholesterol	T cholesterol_HDL_ratio
		Triglycerides	HDL	VLDL
		LDL	Leptin	
	**CBC**	Lymphocytes Absolute	Eosinophils Absolute	Basophils Absolute
		%Lymphocytes	%Neutrophils	Neutrophils Absolute
		WBC Count		
	**Hematology**	Hematocrit	RDW	MCV
		Hemoglobin	RBC Count	Platelet Count
		MCHC	MCH	
	**Health**	Atopic Asthmatics	Metabolic Syndrome	Obese
	**Serum Allergens**	Alternaria Alternata	Cladospor Herbarum	Penicill Notatum
		Aspergill Fumigatus	Dog Dander	Rat Urine Prot
		Cat Dander Epithel	German cockroach	Derm Pter Dustmite
		Derm Farin Dustmite	Mouse Urine Protein	
	**Immunological Markers**	IgECCE	IgGMACA	IgECCE
		IgEMACA	IgGMM1	IgGPCE
		IgEMM2	Ig GMM2	IgGCCE
		IgEPCE	IgGPCE	IgEMACA
GENETIC	**Blood Gene Expression**	AFFY Human U133 Plus 2.0		
	**SNP (11 Genes)**	HLADRB1	IL13	GSTP1
		HLA-DQB1	CD14	GSTT1
		FCER1B	IL4	TNF
		ADAM 33	GSTM1	
BIOMARKER	**Blood metals**	Blood Cu	Blood Se	Blood Hg
		Blood Pb	Blood Zn	
	**Tobacco**	Urinary Hydroxycotinine	Cotinine	Nicotine
	**Nail Metals**	Nail As	Nail Hg	Nail Se
		Nail Cd	Nail Mn	Nail V
		Nail Cr	Nail Pb	Nai Zn
		Nail Se		

	**Urinary Metals**	Urine Se	Urine Zn	Urine Cr
		Urine V	Urine Cd	Urine As
		Urine Cu	Urine Ni_	Urine Pb
		Urine Mn		
	**Urine PAH**	1-OHPHE	1OH-PHE	Sum NAP
		2,3-OHPHE	1OH-PYR	Sum Of All OH
		2OH-FLU	2,3-OHPHE	Sum PHE
		2OH-NAP	2OH-FLU	Creatinine
		9OH-PHE	2OH-NAP	pH
		4OH-PHE	4OH-PHE	Creatinine
		1OH-NAP	9OH-PHE	Mutagenicity (Ames ±S9)
**EXPOSURE**	**Dust Allergen**	Dust Rat	Dust CockroachI	Dust CockroachII
		Dust Mouse		
		Dust Dog_	Dust Cat	Dust Mite (2)
	**Dust Metals**	Dust Cr	Dust Se	Dust Be
		Dust Fe	Dust V	Dust Hg
		Dust Cu	Dust Zn	Dust Pb
		Dust Mn		
	**Dust Mold**	Aspergillus Ustus	Aspergillus Sclerotiorum	Chaetomium Globosum
		Acremonium Strictum	Aspergillus Sydowii	Clad Cladosporioides1
		AsperFlavus_Oryzae	Aspergillus Unquis	Epicoccum Nigrum
		Aspergillus Niger	Aspergillus Versicolor	ERMI_GroupI_GroupII
		Aspergillus Ochraceus	Asper Penicillioides	Eurotium Asp Amstelodami
		Aspergillus Restrictus	Aureobasidium Pullulans	Bac Endotoxin G
		Clad Sphaerospermum	Mold Spore	Mucor Amphibiorum
		Paecilomyces Variotii	Pen Brevicompactum	Pen Corylophilum
		Penicillium Chrysogenum	Penicillium Crustosum	Penicillium Spinulosum
		Penicillium Variabile	Rhizopus Stolonifer	ScopChartarum
		Pen Purpurogenum	ScopBrevicaulis Fusca	TrichodermaViride
		Wallemia Sebi		
	**Dust PAH**	Dust PAH Sum	Benzo(ghi)perylene	Indeno(1,2,3-cd)pyrene
		Benz(a)anthracene	Benzo(k)fluoranthene	Benzo(b)fluoranthene
		Benzo(a)pyrene	Chrysene, iso-Chrysene	Dibenz(a_h)anthracene
	**Indoor/ Outdoor Air Pollution Monitoring**	1,1,1-trichloroethane	Benzene	MTBE
		1,3-Butadiene	Carbon tetrachloride	oxylene
		1,4-dichlorobenzene	Chloroform	Styrene
		2,2,4-Trimethylpentane	Ethylbenzene	Tetrachloroethene
		2,3-Dimethylpentane	Hexane	Toluene
		2-Methylhexane	Methylcyclohexane	3-Methylhexane
		2-Methylpentane	Methylene_Chloride	m,p-xylene

Table 1.
